# ET-GRU: using multi-layer gated recurrent units to identify electron transport proteins

**DOI:** 10.1186/s12859-019-2972-5

**Published:** 2019-07-06

**Authors:** Nguyen Quoc Khanh Le, Edward Kien Yee Yapp, Hui-Yuan Yeh

**Affiliations:** 1 Medical Humanities Research Cluster, School of Humanities, Nanyang Technological University, 48 Nanyang Ave, Singapore, 639798 Singapore; 2 Singapore Institute of Manufacturing Technology, 2 Fusionopolis Way, #08-04, Innovis, Singapore, 138634 Singapore

**Keywords:** Electron transport chain, Cellular respiration, Recurrent neural network, Convolutional neural network, Position specific scoring matrix, Transport protein, Deep learning, Protein function prediction, Gated recurrent units, Web server

## Abstract

**Background:**

Electron transport chain is a series of protein complexes embedded in the process of cellular respiration, which is an important process to transfer electrons and other macromolecules throughout the cell. It is also the major process to extract energy via redox reactions in the case of oxidation of sugars. Many studies have determined that the electron transport protein has been implicated in a variety of human diseases, i.e. diabetes, Parkinson, Alzheimer’s disease and so on. Few bioinformatics studies have been conducted to identify the electron transport proteins with high accuracy, however, their performance results require a lot of improvements. Here, we present a novel deep neural network architecture to address this problem.

**Results:**

Most of the previous studies could not use the original position specific scoring matrix (PSSM) profiles to feed into neural networks, leading to a lack of information and the neural networks consequently could not achieve the best results. In this paper, we present a novel approach by using deep gated recurrent units (GRU) on full PSSMs to resolve this problem. Our approach can precisely predict the electron transporters with the cross-validation and independent test accuracy of 93.5 and 92.3%, respectively. Our approach demonstrates superior performance to all of the state-of-the-art predictors on electron transport proteins.

**Conclusions:**

Through the proposed study, we provide ET-GRU, a web server for discriminating electron transport proteins in particular and other protein functions in general. Also, our achievement could promote the use of GRU in computational biology, especially in protein function prediction.

## Introduction

Proteins accomplish a large diversity of functions inside the various compartments of eukaryotic cells. It is therefore not surprising that protein function prediction is one of the well-studied topics in computational biology and it attracts the attention of numerous researchers conducting their works. There has been a lot of attention given to enhancing the predictive performance of protein functions using a variety of computational techniques. The two most common solutions to address it are namely, finding the best feature sets and using neural networks for prediction. For example, some researchers only used traditional neural networks with a feature set such as position specific scoring matrix (PSSM) [1], biochemical properties [2], and pseudo-amino acid composition (PseAAC) [3]. Nowadays, according to the development of deep learning, many researchers in proteomics have attempted to apply it in predicting protein functions. There have been a lot of works on applying deep neural networks in protein function prediction, such as predicting protein secondary structure [4], efflux protein [5], and Rab GTPases protein [6]. Unfortunately, most of the results have not made full use of the advantages of PSSM profiles in deep neural networks. In all of the previous works, the PSSM profiles have been summed up to a fixed length in order to be fed into neural networks, but in the process, the order information is lost and thus affects performance results. In our work, we wish to present an innovative approach to fill this gap through the incorporation of 1D convolutional neural network (CNN) and gated recurrent unit (GRU). We applied our techniques in the prediction of the function of electron transport protein, which is one of the most essential molecule functions in cellular respiration.

**Fig. 1:**
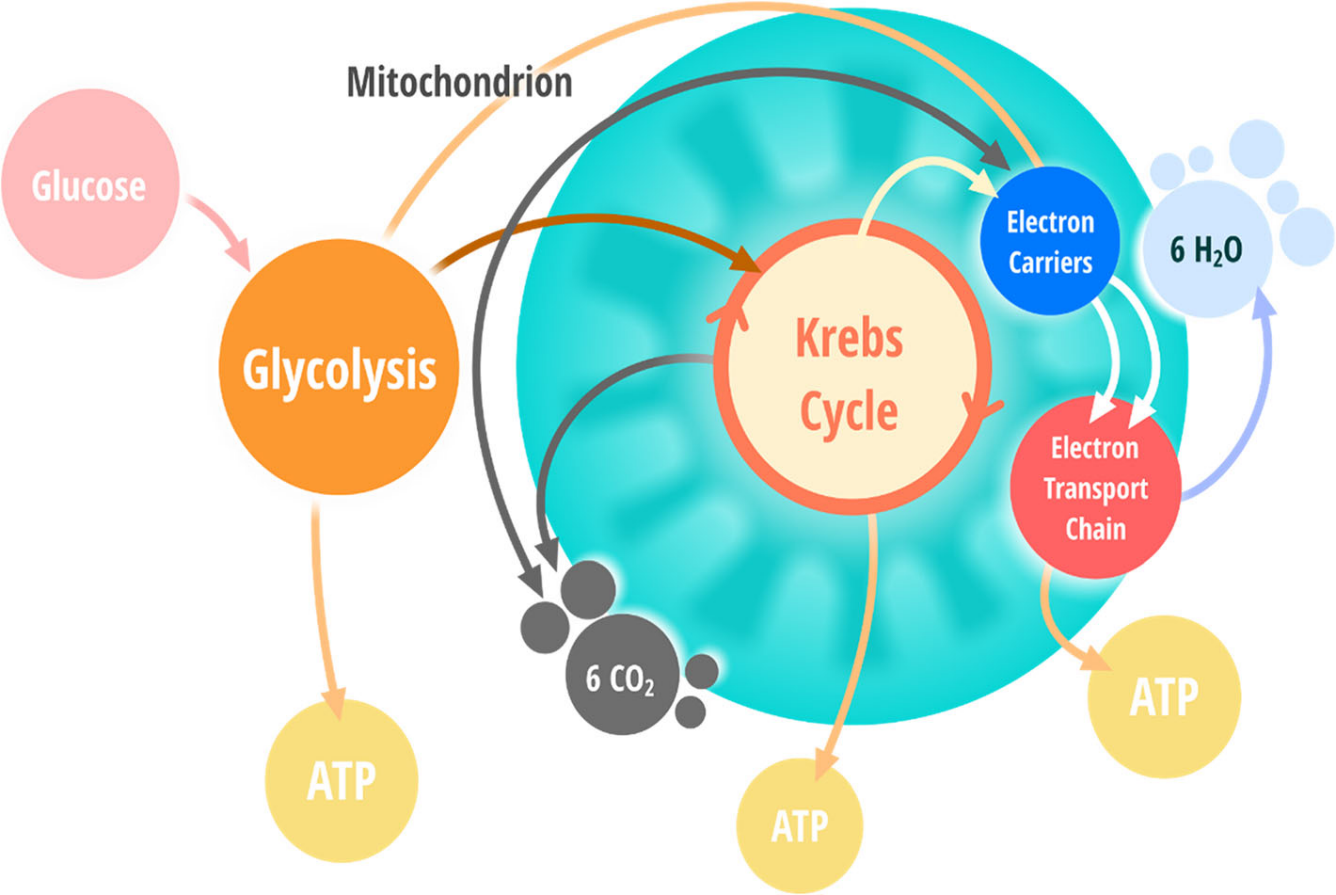
The process of cellular respiration. The goal of cellular respiration is to accumulate electrons from organic compounds to create ATP, which is used to provide energy for most cellular reactions

Cellular respiration is the mechanism for creating adenosine triphosphate (ATP) and it aids cells in obtaining energy from food molecules (i.e. sugar). Figure 1 indicates the process of cellular respiration. The goal of cellular respiration is to accumulate electrons from organic compounds to create ATP, which is used to provide energy for most cellular reactions [7]. An electron transport chain is a pathway to store and transfer electrons in cellular respiration. It can be categorized into five protein complexes: complex I, II, III, IV, and V (ATP Synthase). Each complex consists of different electron carriers and carries out various molecular functions [8]. Electrons donate to complex I from nicotinamide adenine dinucleotide (NADH – a coenzyme found in all living cells) and sequentially pass to complex II, III, IV, and V. During the movement, the hydrogen ions, or protons, pump across the membrane and release water molecules (H2O). Complex V uses the energy created by the pumping process to convert phosphorylate adenosine diphosphate (ADP) to ATP. Numerous types of electron transport proteins have been identified in humans and a series of studies have also indicated that a functional loss of specific complex in electron transport protein resulted in the complication of many diseases [9–13]. Thus, identification of electron transport proteins helps biologists better understand molecular functions and possibly curb the prevalent issue of human disease. Moreover, it is imperative to develop some computational techniques to address the issue.

Recently, there have been some published works on identifying electron transport proteins through the use of computational techniques because of their essential role in cellular respiration, energy production, and human diseases. For instance, one of the most popular studies is Transporter Classification Database (TCDB) [14]. It is a web-accessible, curated and relational database containing the sequence, classification, structural, functional and evolutionary information about transport systems including electron transport proteins from a variety of living organisms. Next, Gromiha [15] discriminated the function of electron transport proteins from membrane proteins using machine learning techniques. According to Chen [16], the transport targets were divided into four types, including electron transporters to do prediction and analysis. In the experiment, they analyze this using amino acid composition (AAC), dipeptide composition (DPC) and PSSM profiles. Mishra et al. [17] also identified electron transport proteins from transport proteins by using PSSM profiles and biochemical properties. Furthermore, Le et al. [18] used radial basis function networks and biochemical properties in identifying the electron transport proteins and their complexes with high accuracy. Le et al. [19] also implemented the ET-CNN, which is a web server that used deep CNN to address this problem with improved accuracy.

Notwithstanding this, previous studies can only be considered as the first step towards a more profound understanding of electron transport proteins. Numerous studies have used PSSM profiles to solve the problem; however, they did not find a solution to input all the PSSM profiles into neural networks. A new approach is therefore needed to address this issue. We thus present a new deep learning architecture which uses 1D CNN, GRU, and PSSM profiles to discriminate electron transport proteins. To our knowledge, no previous computational biology research has specially incorporated the CNN, GRU, and PSSM profiles in identifying protein function. The hybrid of CNN and GRU based recurrent neural network (RNN) has been presented in some sequence-based learning tasks, such as protein secondary prediction [20], discovering complex biological rules to RNA protein-coding potential [21], and quantifying the function of DNA sequences [22]. However, they applied this network on original sequences, which cannot take advantages of advanced biological profiles, (i.e. PSSMs). Here we aim to address this issue.

We documented several key contributions of our study to the field of biology: (1) a new computational model for identifying electron transport proteins which exhibited significant improvements beyond that of previous models, (2) a new deep learning framework constructed from CNN, GRU, and PSSM for classifying the protein functions with high performance, in which we can input all the PSSM profiles into deep neural networks and prevent missing information in PSSM profiles, (3) a benchmark dataset for further study on electron transport protein, and (4) a study that would provide a better understanding of the electron transport protein structures to biologists and researchers through the information collected to aid in any conduction of future research.

## Methodology

**Fig. 2:**
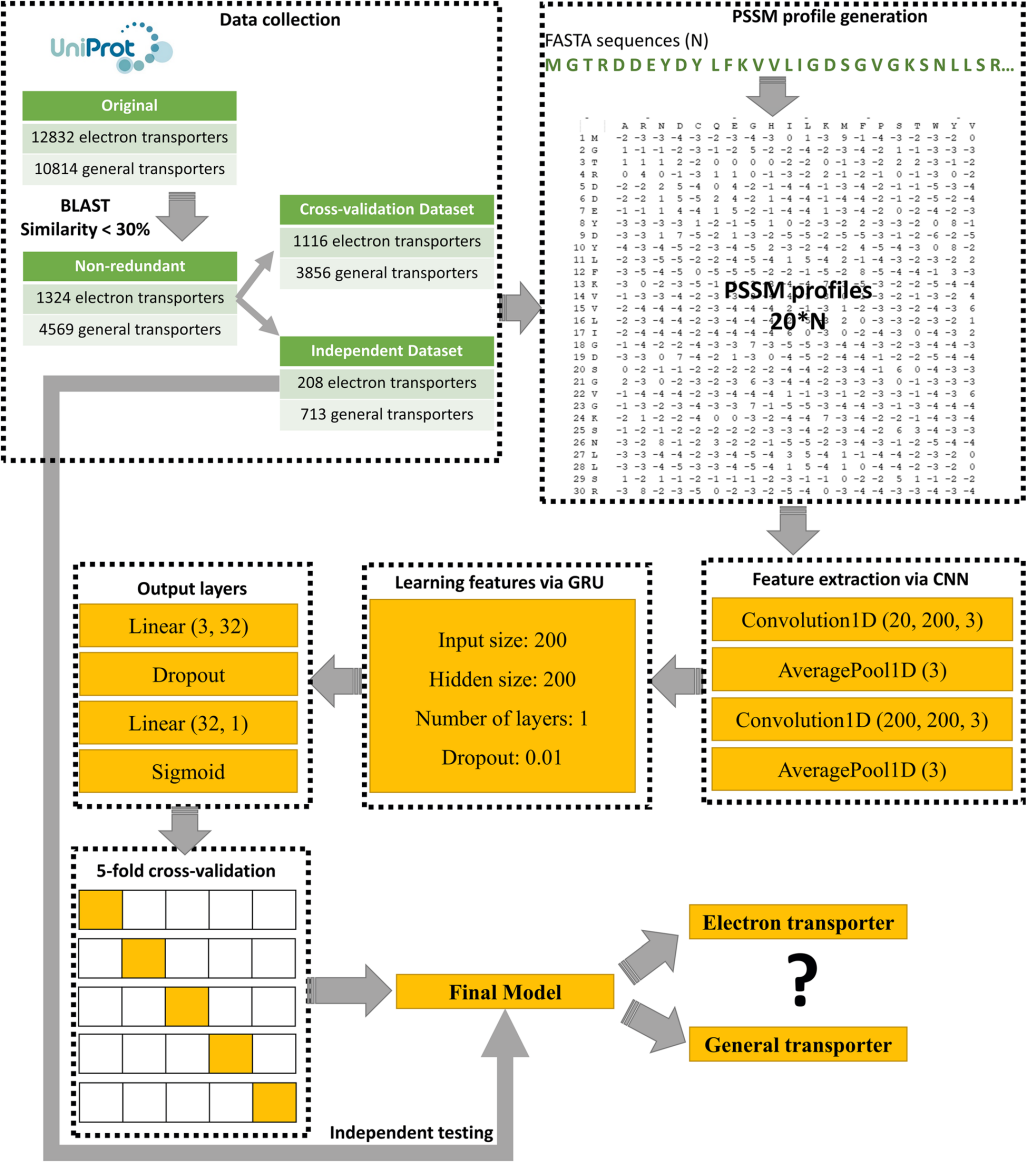
The flowchart for identifying electron transport proteins using 1D RNN, GRU, and PSSM profiles. It included four subprocesses: data collection, feature set generation, neural network implementation and model evaluation

Most experiments have been carried out with a 1D CNN, GRU and PSSM profiles. Figure 2 illustrates a flowchart of the study, and we describe the details of the proposed approach as follows.

### Data collection

In this study, we re-used the benchmark dataset from the previous study [19], which contains 395 electron transport proteins and 2240 transport proteins. However, to take full advantage of deep learning, we collected more data from UniProt release-2018_05 (on 23-May-2018) [23] and Gene Ontology (GO) release-2018-05-01 [24], which provide high quality resources for research on gene products. Notice that we only chose the sequences which have been reviewed by scientists in their published papers. After this step, we received 12,832 electron transport proteins and 10,814 general transport proteins in all of species. Subsequently, BLAST (version 2.2.26) [25] was applied to remove the redundant sequences with identities that have a similarity of more than 30%. The data collected also reveals that the rest of the proteins can be divided into 1324 electron transport proteins and 4569 general transport proteins. This step aims to prevent overfitting in our model. Labelling the proposed issue as a binary classification problem, we solved it by using electron transport proteins as the positive data and the general transport proteins as the negative data. To conduct the experiments, we needed two sets of data: cross-validation and independent datasets. In these two datasets, the independent dataset contained newly discovered proteins and the cross-validation dataset contained the rest of the data. The cross-validation dataset was used for constructing our model, and the independent dataset was used for evaluating the performance of the proposed method. Table 1 lists all the detail of the dataset in this study.

**Table 1:** Statistics of all retrieved electron transport proteins and general transport proteins

	Original	Similarity < 30%	CV	IND
Electron transport	12,832	1324	1116	208
General transport	10,814	4569	3856	713

*CV* Cross-validation, *IND* Independent

### Encoding feature sets from the protein sequence information

In this study, the feature extraction method that is being applied is PSSM, a matrix represented by all the amino acid patterns in protein sequences. The matrix is also used for decoding the evolutionary information of a protein sequence. PSSM was first presented by Jones [1] and increasingly applied to many bioinformatics applications with significant improvements [26–28]. To generate the PSSM profiles from FASTA sequences, we used PSI-BLAST [25] to search against non-redundant protein database for two iterations. The query to produce the PSSM profile is as follows:

*psiblast.exe -num_iterations 2 -db < nr > −in_msa < fasta_file > −out_ascii_ < pssm_file>.*

Numerous studies have attempted to identify the protein function by using PSSM and the summing method [18, 26, 29]. This means that they tried to sum up the same amino acids and convert PSSM profiles with a 20*N matrix to a 20*20 matrix. This method helped them to fix the input length to insert into neural networks. However, this also raises the big issue of the loss of information when PSSM profiles are in order. Therefore, in this study, we attempted to input all PSSM profiles into deep neural networks via GRU architectures. We also ensured that the position information was not lost and we were able to preserve this information while sequences of different lengths remain comparable.

### Feature extraction using one-dimensional convolutional neural networks

Our deep learning architecture to identify electron transport protein contains 1D CNN to extract the features, and GRU to learn the features in order to build models. This is a deep neural network architecture which had been shown to handle sequential data significantly in various fields. Different from traditional neural network, RNN can take advantage of sequence information and theoretically, it can utilize the information of arbitrary length sequence. It is able to memorize parts of the inputs and use them to make accurate predictions. Our deep neural network structure was implemented using PyTorch library [30]. We also accelerated the performance via graphic processing unit (GPU) computing and CUDA kernel. The input is a multiplication of sequence length and size of amino acid vocabulary. The CNN extracts amino acid information using various filter sizes. To extract the PSSM information, we first applied a 1D convolution over an input shape consisting of several input matrices. The convolutional layer takes a sliding window that is moved in stride across the input, transforming the values into representative values. During this process, convolution operation preserves the spatial relationship between numeric values in the PSSM profiles by learning useful features using small squares of input data [31]. Each protein was treated as a separate sample and input to the neural network. Given an input size (N, C_in_, L), we can precisely compute the output (N, C_out_, L_out_) by using the following formula:

$$ out\left({N}_i,{C}_{out_j}\right)= bias\left({C}_{out_j}\right)+\sum \limits_{k=0}^{C_{in}-1} weight\left({C}_{out_j},k\right)\ast input\left({N}_i,k\right)\kern1.75em (1) $$

where * is the valid cross-correlation operator, N is the batch size, C denotes the number of channels, and L is the length of the signal sequence. In this study, the input is the sequence length multiplied by the size of the amino acid vocabulary (=20). The big advantage of inputting all the PSSM features into the neural network is to prevent missing information from PSSM profiles. We also set the learnable weights and bias variables of the module of shape. The pooling layer is usually inserted between the convolutional layers with the aim of reducing the size of matrix calculations for the next convolutional layer. The operation performed by this layer is also called “down-sampling” as it removes certain values leading to less computational operations and overfitting control while still preserving the most relevant representative features. The pooling layer also takes a sliding window or a certain region that is moved in stride across the input matrix transforming the values into representative values [31]. In our study, we performed a 1D average pooling over an input of several values. In this step, we can also calculate the output (N, C, L) and kernel size k as follows:

$$ out\left({N}_i,{C}_j,l\right)=\frac{1}{k}\sum \limits_{m=0}^k input\left({N}_i,{C}_j, stride\ast l+m\right)\kern0.75em (2) $$

Zero-padding is the process of symmetrically adding zeros to the input matrix which allows the size of the input to be adjusted to certain requirements. In the model presented in the current study, zero values were added at the beginning and end of the matrices. This allowed us to apply the filter to the border positions of the matrices. If padding is non-zero, then the input is implicitly zero-padded on both sides for padding number of points. The input shape (N, C, L_in_) and output shape (N, C, L_out_) can be calculated by:

$$ {L}_{out}=\left\lfloor \frac{L_{in}+2+ padding- kernel\_ size}{stride}+1\right\rfloor \kern0.75em (3) $$

### Learning and classification using GRU

After generating feature sets with 1D CNN, we applied a multi-layer GRU to an input sequence. GRU, with its so-called update gate and reset gate, is an improved version of the standard recurrent neural network (RNN). Because of the problem of “vanishing gradient” in the network structure, RNN can only retrospectively utilize the information on time steps which are close to it in practical applications. In order to solve this problem, Long Short Term Memory (LSTM) and GRU were presented with specially designed network architecture, which can learn long-term dependencies information naturally. Basically, these are two vectors which decide what information should be passed to the output. The special thing about them is that they can be trained to keep old or previous information, without removing information that is irrelevant to the prediction. The idea behind a GRU layer is quite similar to that of a LSTM layer, as are the equations. For each element in the input sequence, each layer computes the following function:

(1) Update gate helps the model to determine how much of the past information (from previous time steps) needs to be passed along to the future. We calculated the update gate *z*_*t*_ for time step *t* using the formula:

$$ {z}_t=\sigma \left({W}_{iz}{x}_t+{b}_{iz}+{W}_{hz}{h}_{\left(t-1\right)}+{b}_{hz}\right)\kern1.25em (4) $$

where *x*_*t*_ is the input at time *t*, *h*_*(t − 1)*_ is the hidden state of the previous layer at time *t-1* or the initial hidden state at time 0, *σ* is the sigmoid function, *W* is the weight, and *b* is the bias.

(2) Reset gate is used from the model to decide how much of the past information is forgotten. To calculate it, we use:

$$ {r}_t=\sigma \left({W}_{ir}{x}_t+{b}_{ir}+{W}_{hr}{h}_{\left(t-1\right)}+{b}_{hr}\right)\kern1.25em (5) $$

(3) Current memory content uses the reset gate to store the relevant information from the past:

$$ {n}_t=\mathit{\tanh}\left({W}_{in}{x}_t+{b}_{in}+{r}_t\left({W}_{hn}{h}_{\left(t-1\right)}+{b}_{hn}\right)\right)\kern1.25em (6) $$

(4) Final memory at current time step: as the last step, the network needs to calculate *h*_*t*_ vector which holds information for the current unit and passes it down to the network. In order to do that the update gate is needed. That is done as follows:

$$ {h}_t=\left(1-{z}_t\right){n}_t+{z}_t{h}_{\left(t-1\right)}\kern1.25em (7) $$

### Output layers

In output layers, we used two linear layers to apply linear transformation to the incoming input. The first layer with input feature size (= GRU hidden size) and output feature (= fully connected layer size) aims to transform the output of GRU layers. Then the next linear layer was applied with input feature size of fully connected layer size and output size of 1 to generate the output results of the model. Noted that we set bias = true in this step to let the layer learn the additive biases.

The next layer applied was dropout, which is an effective technique for the regularization and prevention of the co-adaptation of neurons as described in the paper [32]. The importance of the dropout layer was to enhance the predictive performance of our model and prevent the problem of overfitting. In the dropout layer, the model will randomly deactivate the neurons in a layer that have a certain probability value. If the dropout value is tuned to a layer, the neural network will learn different, redundant representations, and the training time will be faster. In this study, the dropout values are floats ranging from 0 to 1 to evaluate our model.

The last element in the output layers is sigmoid, which is a non-linear activation. Commonly, sigmoid function is problematic in RNN and it applies the element-wise function as follows:

$$ Sigmoid(x)=\frac{1}{1+\mathit{\exp}\left(-x\right)}\kern2.5em (8) $$

### Assessment of predictive ability

We used five-fold cross-validation to evaluate the performance of ET-GRU and the comparison model. In each validation, all data randomly divides into five equal parts. Four-fold set data are taken as train data, the rest one-fold is taken as test data. To guarantee the unbiased comparison, it confirmed that there is no overlap between train data and test data. Because 5-fold cross-validation will yield different results each time, we implemented 10 iterations of 5-fold cross-validation and averaged the results across the 10 iterations. Furthermore, to control for any systematic bias in the cross-validation set, the independent dataset was used to evaluate the performance accuracy. We followed the widely used evaluation criteria in many bioinformatics studies [5, 33, 34]. Some standard metrics were used, such as sensitivity (Sen), specificity (Spe), accuracy (Acc) and Matthews correlation coefficient (MCC) using the formula presented in those studies.

$$ Sen sitivity=1-\frac{N_{-}^{+}}{N^{+}},\kern0.5em 0\le Sen\le 1\kern3.5em (9) $$

$$ Spec ificity=1-\frac{N_{+}^{-}}{N^{-}},\kern0.5em 0\le Spec\le 1\kern3.5em (10) $$

$$ Acc uracy=1-\frac{N_{-}^{+}+{N}_{+}^{-}}{N^{+}+{N}^{-}},\kern0.5em 0\le Acc\le 1\kern3em (11) $$

$$ MCC=\frac{1-\left(\frac{N_{-}^{+}}{N^{+}}+\frac{N_{+}^{-}}{N^{-}}\right)}{\sqrt{\left(1+\frac{N_{+}^{-}-{N}_{-}^{+}}{N^{+}}\right)\left(1+\frac{N_{-}^{+}-{N}_{+}^{-}}{N^{-}}\right)}},\kern1em -1\le MCC\le 1\kern2.75em (12) $$

The relationship between these symbols in Eqs. (9, 10, 11 and 12) are indicated by:

$$ \left\{\begin{array}{c}{N}_{+}^{-}= FP\\ {}{N}_{-}^{+}= FN\\ {}\begin{array}{c}{N}^{+}= TP+{N}_{-}^{+}\\ {}{N}^{-}= TN+{N}_{+}^{-}\end{array}\end{array}\right.\kern8.75em (13) $$

where TP means the true positives and refers to the number of electron transport proteins that were correctly predicted by the classifier, TN means true negatives and refers to the number of general proteins that were correctly predicted by the classifier, FP means false positives and refers to the number of electron transport proteins that were incorrectly predicted by the classifier, and FN means false negative and refers to the number of general proteins that were incorrectly predicted by the classifier.

## Results

### Composition amino acid of electron transport proteins and general transport proteins

**Fig. 3:**
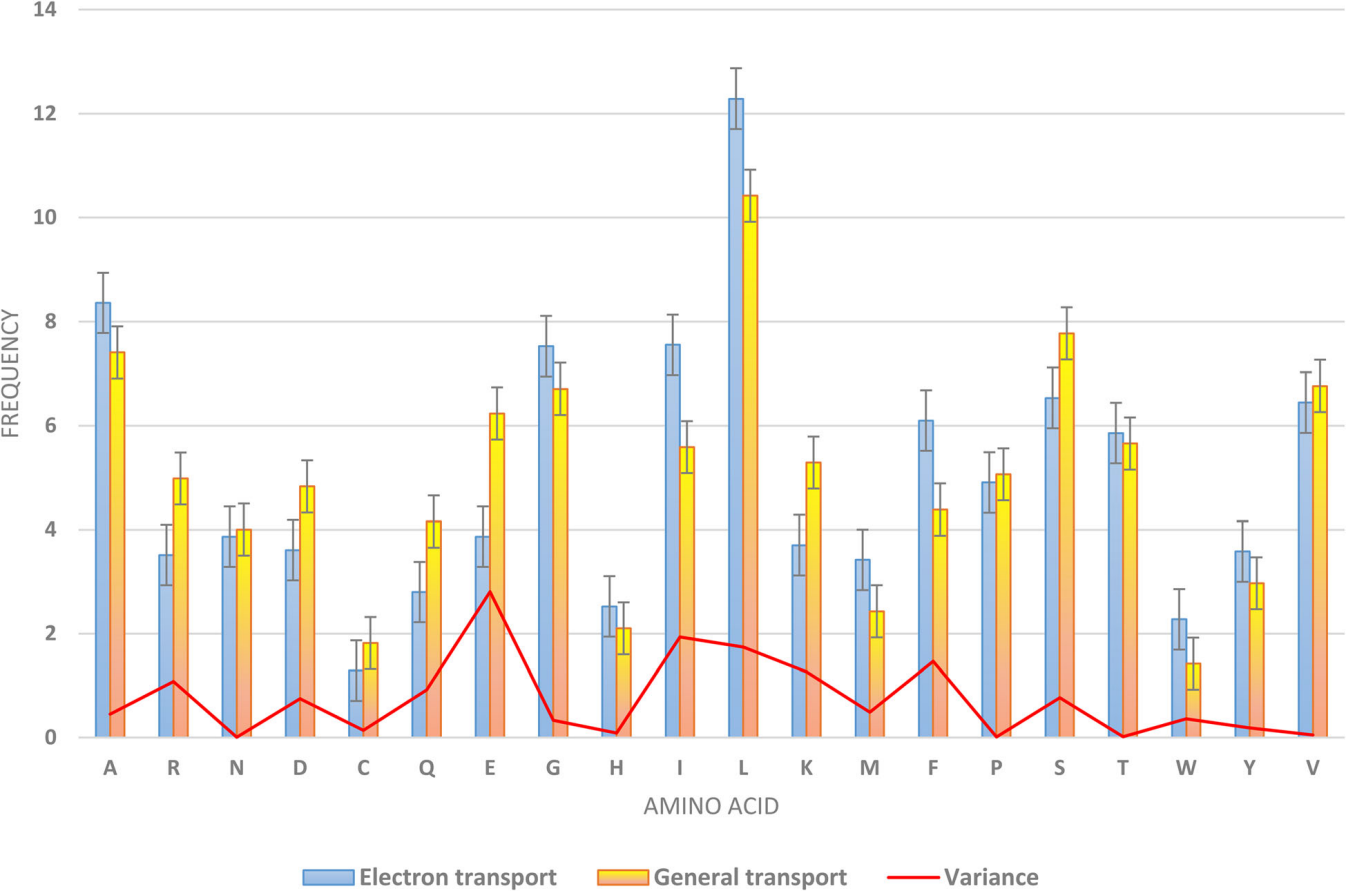
Amino acid composition and variance of amino acid composition in electron transport and general transport proteins. There are numerous differences between the amino acid frequencies surrounding the electron transport proteins and general transport proteins. For instance, the amino acid E, I, F, or R could be adopted for classifying electron transport proteins

We analyzed the composition of amino acid in electron transport proteins and general transport proteins by computing the frequency between them. Figure 3 illustrates the amino acids which contributed the highest frequency in two distinct datasets. We realized that there were numerous differences in amino acid frequencies between those surrounding the electron transport proteins and those surrounding the general transport proteins. For instance, the amino acid E, I, L, or F (with higher variances) could be applied for classifying electron transport proteins. Also, we used the standard error bars on the chart to show the significant differences in the contributions of these amino acids. If two error bars do not overlap, we can conclude that the difference between these two datasets is statistically significant. In Fig. 3, it is easy to say that there are significant differences between electron transporters and general transporters in amino acid R, D, Q, E, I, L, K, F, and S. Thus, these amino acids distributions certainly possessed an essential role in discriminating electron transport proteins from general proteins.

### Model selection

We have classified the electron transport proteins using various hyper-parameters i.e., convolutional feature size, kernel size, fully connected layer size, and so on. First, we used six filters ranging from 32 to 1024 to train the cross-validation and in the consideration of the optimal filter. The fully connected layer size of 32 achieved the highest performance in discriminating the electron transport proteins with an accuracy of 93.5 and 92.3% for the cross-validation and independent datasets, respectively. This point proved that a bigger filter size did not have a significant impact to this problem, and thus we only need to use the simplest filter size while still be able to achieve significant results. Comparing these results with those of the other performances, we also saw that the result was consistent between cross-validation and independent datasets. It means that the hyper-parameters from the cross-validation test can be used to evaluate the independent test with the same level of performance. Our model did not run into the overfitting problem either. Further, we also examined the performance results of different GRU hidden layer sizes. For these experiments, the GRU hidden layer size of 200 performed better than others. In summary, Table 2 shows all layers with weights and trainable parameters of our GRU model. Thereafter, we decided to use these parameters for the rest of the experiments.

**Table 2:** All layers with weights and trainable parameters in the proposed method

Layer	Weights	Parameters
Conv1d (20, 200, 3)	((200, 20, 3), (200,))	12,200
AvgPool1d (3)	0	0
Conv1d (200, 200, 3)	((200, 200, 3), (200,))	120,200
AvgPool1d (3)	0	0
GRU (200, 200, 1)	((600, 200), (600, 200), (600,), (600,))	241,200
Linear (200, 32)	((32, 200), (32,))	6432
Dropout (0.5)	0	0
Linear (32, 1)	((1, 32), (1,))	33
Sigmoid ()	0	0

### Comparative performance between the proposed method and the previous technique on PSSM profiles

From the model selection (previous section) we identified the optimal hyper-parameters for the best performing model architecture. We then compared our performance with those of previous state-of-the-art techniques. Previous techniques on PSSM profiles summed up all of the same amino acids to become a vector 400D or a matrix 400D to input into neural networks. This technique was efficiently used in a variety of bioinformatics applications and achieved significant results [16, 19]. With this technique, the composition of amino acids will be kept as the features in networks. However, the order information is missing and our method fills the gap of this missing information. Due to the significant improvements of k-nearest neighbour (kNN) [35], Random Forest [36], and support vector machine (SVM) kernel [37] in many bioinformatics applications, we conducted experiments using these classifiers. The next classifier that we would like to conduct experiments for comparison is CNN, which is currently considered as the best method for this type of problem [19]. All the processes for tuning parameters had been carried out on the training dataset and the optimizations had been chosen according to the accuracy metric. We varied the number of nearest neighbors in kNN from one to ten (step size of 1), performed a grid search to estimate the accuracy of each parameter combination to find the optimal cost and gamma in LibSVM (log2c was ranged from − 5 to 5 (step size of 2), log2g was ranged from − 4 to 0 (step size of − 2)), number of trees were ranged from 100 to 500 (step size of 100) in RandomForest; and hyper-parameter tuning in CNN. The optimal parameters of these classifiers are shown in Table 3’s footnote.

**Table 3:** Predictive performance of classifying electron transport proteins using different neural networks

	CV				Independent			
	Sen	Spe	Acc	MCC	Sen	Spe	Acc	MCC
kNN	37.7(−)	98.9(+)	85.2(−)	0.53(−)	32.7(−)	96.5(+)	82.1(−)	0.41(−)
RF	64.8(−)	97.1(+)	89.8(−)	0.69(−)	56.3(−)	96.4(+)	87.3(−)	0.61(−)
SVM	74(−)	96.2(+)	91.2(−)	0.74(−)	74(−)	91.7(−)	87.7(−)	0.65(−)
CNN	73.8(−)	95(−)	90.3(−)	0.71(−)	78.2(+)	92.5(−)	89.5(−)	0.69(−)
GRU	83.7	96.3	93.5	0.81	79.8	95.9	92.3	0.77

*Note:* (kNN: k = 10, RF: n_estimators = 500, SVM: c = 32, g = 0.125, CNN: 128 filters, GRU: 32 filters, (+) for significantly better than GRU, (−) for significantly worse than GRU in a two-proportion z-test)

**Fig. 4:**
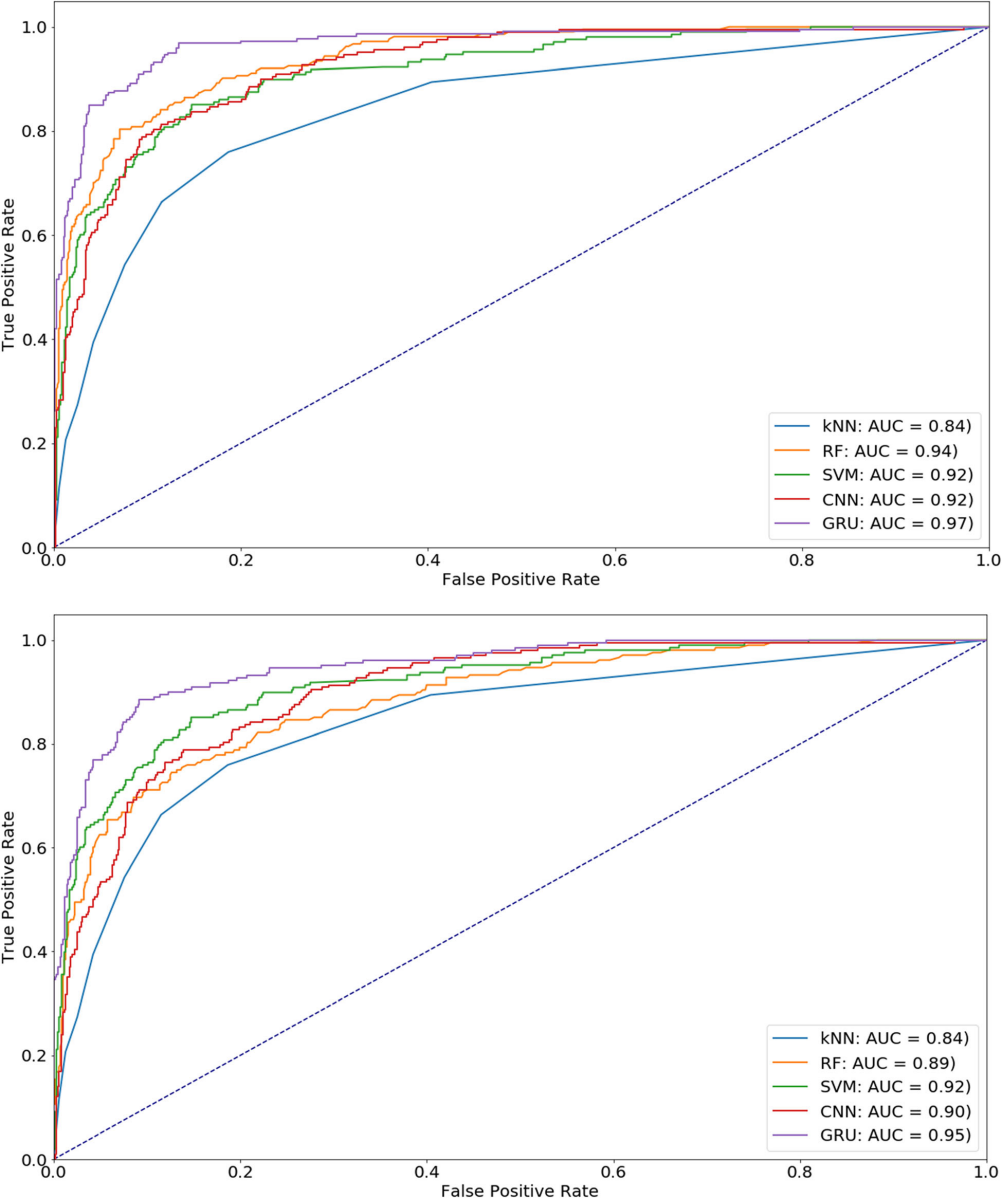
ROC Curves for predicting electron transport protein using GRU and PSSM profiles. (**a**) cross-validation testing, (**b**) independent testing

Table 3 shows the comparative performance between the proposed method, GRU, with previous methods on the same dataset. We used a two-proportion z-test to determine whether other methods are significantly better (+), worse (−) or have no statistical difference compared with GRU at a confidence level of 95%. As shown in Table 3, the statistical tests show that GRU exhibited higher performance than other techniques for most of the given evaluation metrics. Furthermore, to have a more comprehensive and intuitive assessment of prediction models, ROC Curves are provided in this section. As shown in Fig. 4, the Area Under the Curve (AUC) of our proposed method (GRU) outperformed the other methods (AUC = 0.97 and 0.95 in the cross-validation and independent tests, respectively). Therefore, many evidence supported us to claim that the order information of PSSM plays an important role in classifying the protein function in general and electron transport in particular.

### Benchmark PSSM profiles with different sensitive alignment methods

As shown in some of the previous publications in bioinformatics [38, 39], sequence alignments were easily applicable to any protein class recognition. Therefore, we aim to benchmark the proposed method with other sensitive alignment methods. We consulted the previous work from [38] to generate different alignments such as PSI-BLAST and hidden Markov model (HMM). PSI-BLAST alignment has been done with BLAST package and e-value of 0.01. As shown in BLAST manual, BLAST hit with e-value smaller than 0.01 can be considered as a good hit for homology matches and it can be used to compare with our PSSM profiles. Another profile that we would like to compare is the HMM profile, which is a probabilistic model that encapsulates evolutionary changes that have occurred in a set of biological sequences. To generate HMM profiles, we used Pfam as our database for scanning. Table 4 shows the comparative performance among different alignment methods in the independent test. It is observed that the PSSM profile was superior to the other methods in most of the metrics. Therefore, we can again claim that this network architecture is useful for generating the hidden information of PSSM profiles.

**Table 4:** Predictive performance of classifying electron transport proteins using different sensitive multiple alignments

	Sen	Spe	Acc	MCC
PSI-BLAST	75.5	83.6	81.8	0.54
HMM	76	95	90.8	0.73
PSSM	79.8	95.9	92.3	0.77

### Comparison to current predictors on electron transport proteins

To ensure a new approach is efficient and fair, we need to make a comparison with the predictions from other published works on the same dataset. In this section, we would like to compare our work with some of the recently published works on electron transport proteins: TrSSP [17], Chen et al. [16], Le et al. [18], and ET-CNN [19]. Table 5 shows the comparison between our proposed method and other predictions on the cross-validation dataset. We easily observed that our method performed better than the others for any given evaluation metric. However, the comparison is not sufficiently fair because most of works used a different dataset. They are only considered as a relative comparison and need a more accurate and fair comparison.

**Table 5:** Comparison with state-of-the-art predictions on the cross-validation dataset and independent dataset

	Sen	Spe	Acc	MCC
Cross-validation				
TrSSP [17]	85	80	81.43	0.6
Chen et al. [16]	71.6	93.5	90.1	0.62
Le et al. [18]	74.6	95.8	92.9	0.7
ET-CNN [19]	51.1	96.1	89.4	0.54
ET-GRU	83.7	96.3	93.5	0.81
Independent				
ET-CNN [19]	52.9(−)	96.6(+)	86.8(−)	0.59(−)
ET-GRU	79.8	95.9	92.3	0.77

with ET-GRU as the base case, (+) and (−) indicates whether ET-CNN is significantly better or worse, respectively

Therefore, to have a fair comparison, we attempted to apply the independent test to evaluate how they perform. At this step, we chose ET-CNN [19] as a basis of comparison because a web server is provided and it is also the latest one for this type of data. As shown in Table 5, ET-GRU is able to predict electron transport proteins with higher sensitivity, accuracy, and MCC. This higher performance is evidence of identifying electron transport proteins with higher accuracy than previous techniques. Therefore, we are able to conclude that the order information plays an important role in PSSM profiles and our approach can help identify electron transport proteins more accurately.

### Web server development

To allow readers and users to assess the proposed method, we provided a simple web server which can be freely accessible at http://140.138.155.216/etgru/. The implementation of ET-GRU was done by Python language and Flask framework. ET-GRU can be used by a wide variety of biologists with no knowledge of computational techniques. The users only need to submit the amino acid sequence(s) in the ‘FASTA’ format. Our server then processes all the submitted sequences and predicts them. The best model was integrated into our web server which helps the users identify their sequence belongs to electron transport proteins or not.

## Discussions

In this study, we constructed a new dataset for identifying electron transport proteins from general transport proteins and there are a few differences between our dataset and previous dataset [19]. In the ET-CNN dataset, the amino acids playing important roles in the electron transport protein are A, S, and G while our dataset can provide some additional contributions from amino acid E, I, L, or F. Therefore, our classifiers are able to capture more information to classify and reach high performance results. Further, the more data collected, the more differences between the two types of dataset in the composition of amino acids.

Furthermore, feature extractions played an essential role in discriminating protein functions in general and electron transport protein in particular. Although a number of methods have been proposed for extracting features of protein sequences via PSSM profiles [16, 18, 19], most of them showed great limits on ordering information. ET-GRU is able to prevent this missing information in PSSM via a combination of 1D CNN and GRU. As a result, it was superior to the previous techniques on PSSM profiles which has been applied successfully in a lot of bioinformatics applications. We also compared our performance with the previous works [15, 16, 18, 19] and our ET-GRU also outperformed the others on the same dataset. It is strong evidence that ordering information of PSSM profiles plays a vital role in improving predictive performance. Another reason is the use of deep neural network which helped to extract hidden information in PSSM profiles better than other shallow networks.

However, our study still endures some limitations and there remain possible approaches to enhance the performance results in the future. Firstly, a bigger dataset needs to be retrieved and used to get full advantage of deep learning. Secondly, future studies could investigate how to integrate the PSSM information and the other state-of-the-art features (i.e., biochemical properties, physicochemical properties, or pseudo components) to maximize the performance of GRU network. Thirdly, there is necessary for retrieving a standard negative dataset instead of using general transport proteins.

## Conclusions

This paper has proposed an innovative method using 1D CNN, GRU, and PSSM profiles for discriminating the electron transport proteins. This is the first study that has applied this method to protein function prediction. With this method, we are able to preserve all of the PSSM information which is fed into the deep neural networks. We evaluated its performance using 10 iterations of 5-fold cross-validation and an independent test dataset (208 electron transport proteins and 713 general transport proteins). Our method showed an average 5-fold cross-validation accuracy of 93.5% and MCC of 0.81 for predicting electron transport proteins. The accuracy and MCC with the independent dataset are 92.3% and 0.77, respectively. Compared with the performance of the state-of-the-art predictors, this approach achieved an evinced improvement in all of the measurement metrics. Throughout this study, we experimented with a powerful model that identifies the new proteins that are electron transport proteins with high accuracy. The findings of this study act as a potential basis for further research that can use the combination of CNN, GRU, and PSSM profiles in bioinformatics. Moreover, scientists can use our approach to solve various protein function prediction problems in the future.

## Data Availability

The datasets generated and/or analyzed during the current study are available in the GitHub repository, https://github.com/khanhlee/et-gru.

## Abbreviations

AAC: Amino acid composition
ADP: Adenosine diphosphate
ATP: Adenosine triphosphate
AUC: Area Under The Curve
CNN: Convolutional neural network
DPC: Dipeptide composition
GRU: Gated recurrent units
kNN: k-nearest neighbor
LSTM: Long Short Term Memory
MCC: Matthew’s correlation coefficient
NADH: Nicotinamide adenine dinucleotide
PSSM: Position specific scoring matrix
RNN: Recurrent neural network
ROC: Receiver operating characteristic
SVM: Support vector machine
TCDB: Transporter Classification Database
